# Wnt16 Increases Bone-to-Implant Contact in an Osteopenic Rat Model by Increasing Proliferation and Regulating the Differentiation of Bone Marrow Stromal Cells

**DOI:** 10.1007/s10439-024-03488-y

**Published:** 2024-03-22

**Authors:** Michael B. Berger, Kyla Bosh, Jingyao Deng, Thomas W. Jacobs, D. Joshua Cohen, Barbara D. Boyan, Zvi Schwartz

**Affiliations:** 1 Department of Biomedical Engineering, College of Engineering, Virginia Commonwealth University, 601 West Main Street, Richmond, VA 23284 USA; 2 Wallace H. Coulter Department of Biomedical Engineering at the Georgia Institute of Technology and Emory University, 313 Ferst Drive, Atlanta, GA 30332 USA; 3 Department of Periodontology, University of Texas Health Science Center at San Antonio, 7703 Floyd Curl Drive, San Antonio, TX 78229 USA

**Keywords:** Titanium, Surface properties, Bone marrow stromal cells, MSCs, Cortical bone, Botox

## Abstract

Osseointegration is a complex biological cascade that regulates bone regeneration after implant placement. Implants possessing complex multiscale surface topographies augment this regenerative process through the regulation of bone marrow stromal cells (MSCs) that are in contact with the implant surface. One pathway regulating osteoblastic differentiation is Wnt signaling, and upregulation of non-canonical Wnts increases differentiation of MSCs on these titanium substrates. Wnt16 is a non-canonical Wnt shown to regulate bone morphology in mouse models. This study evaluated the role of Wnt16 during surface-mediated osteoblastic differentiation of MSCs in vitro and osseointegration in vivo. MSCs were cultured on Ti substrates with different surface properties and non-canonical Wnt expression was determined. Subsequently, MSCs were cultured on Ti substrates +/−Wnt16 (100 ng/mL) and anti-Wnt16 antibodies (2 μg/mL). Wnt16 expression was increased in cells grown on microrough surfaces that were processed to be hydrophilic and have nanoscale roughness. However, treatment MSCs on these surfaces with exogenous rhWnt16b increased total DNA content and osteoprotegerin production, but reduced osteoblastic differentiation and production of local factors necessary for osteogenesis. Addition of anti-Wnt16 antibodies blocked the inhibitor effects of Wnt16. The response to Wnt16 was likely independent of other osteogenic pathways like Wnt11-Wnt5a signaling and semaphorin 3a signaling. We used an established rat model of cortical and trabecular femoral bone impairment following botox injections (2 injections of 8 units/leg each, starting and maintenance doses) to assess Wnt16 effects on whole bone morphology and implant osseointegration. Wnt16 injections did not alter whole bone morphology significantly (BV/TV, cortical thickness, restoration of trabecular bone) but were effective at increasing cortical bone-to-implant contact during impaired osseointegration in the botox model. The mechanical quality of the increased bone was not sufficient to rescue the deleterious effects of botox. Clinically, these results are important to understand the interaction of cortical and trabecular bone during implant integration. They suggest a role for Wnt16 in modulating bone remodeling by reducing osteoclastic activity. Targeted strategies to temporally regulate Wnt16 after implant placement could be used to improve osseointegration by increasing the net pool of osteoprogenitor cells.

**Supplementary Information:**

The online version contains supplementary material available at 10.1007/s10439-024-03488-y.

## Introduction

Dental and orthopedic implants are used to restore function and improve quality of life for patients after bone fracture, traumatic damage, or impaired bone quality from a variety of bone metabolic disorders [10, 21, 61]. Implant designs and manufacturing processes are tailored to the anatomical location needing restoration. Screw style implants in the mouth, cages in the spine, and stems in hip arthroplasties are specifically tailored to increase the initial stability of the load sustaining implant. This initial stability is vital to the long-term success of the implant and is dependent on cortical bone thickness, which is decreased in most cases requiring implant treatment. Therefore, there is a clinical need to develop implant technologies that work to increase the cortical bone thickness adjacent to an implant to promote implant stability and biological osseointegration [10, 12, 21, 27, 41, 61, 62].

While initial stability can be achieved through the macroscale design features of an implant, long-term stability requires successful osseointegration. Osseointegration is the direct and functional connection of healthy bone tissue to a synthetic biomaterial and is comprised of a multi-stage complex biological cascade requiring multipotent progenitor cells, immune cells, and differentiated bone forming osteoblasts [1, 5, 26, 60]. Surface properties have been shown to tightly regulate and affect osseointegration in healthy and compromised bone. Two of these properties, microscale roughness and wettability, have been extensively studied to determine the role of each in the overall cellular response of cells that are within the peri-implant environment during osseointegration [10, 20, 24].

Titanium and Ti alloys are used in these bone-facing implants due to their high resistance to corrosion and wear, as well as their biocompatibility properties, which are ascribed to a passivating oxide layer (TiO_2_) on the surface [12, 27, 41, 62]. A multiscale microtopography on Ti implants has been shown to augment the differentiation of bone marrow stromal cells (MSCs) into bone forming osteoblasts through a process termed surface-mediated osteoblastic differentiation [8]. Surface wettability further augments the effect of the surface topography, increasing the production of osteogenic factors necessary for bone formation. On Ti surfaces possessing irregular triangle-like structures at the micron, submicron, and nanoscale, MSCs alter membrane receptor profiles, including upregulating receptors for bone morphogenetic protein 2 (BMP2) [49], and differentiate into osteoblasts. MSCs switch from expressing fibronectin-binding α5,β1 integrins, used during proliferation and migration, to collagen-binding α2,β1 heterodimers [28, 39, 51]. These changes are also correlated with increased expression of proteins associated with an osteoblast phenotype including osteocalcin (OCN), osteopontin (OPN), runt-related transcription factor 2 (RUNX2), and osteoprotegerin (OPG). The MSCs change shape, pulling away from the surface and a spindle fiber morphology into a more suspended and columnar osteoblast shape [31, 33, 36]. In addition, they produce factors involved in osteogenesis, including BMP2 and BMP4, transforming growth factor beta 1 (TFGβ1), vascular endothelial growth factor-165 (VEGF), basic fibroblast growth factor (FGF2), and semaphorin 3A [40, 42, 53]. Factors that modulate osteoclast recruitment and activity, such as OPG, OPN and semaphorin 3C (sema3C) are upregulated as well [29, 43].

Non-canonical Wnt signaling plays a regulatory role in this differentiation pathway and is important for bone formation adjacent to an implant surface. Non-canonical Wnts use small intracellular molecules and calcium ions to transduce signals from outside the cell to the nucleus. In this family, Wnt5a has been shown to be upregulated and secreted during osteoblastic differentiation on Ti substrates in a surface topography-dependent manner [15, 50]. The increased production of Wnt5a results in increased gene expression of RUNX2 and production of BMP2 and BMP4, as well as VEGF and OPG. Experiments evaluating specific inhibitors and blocking antibodies of Wnt5a and non-canonical signaling have shown specificity of this signaling motif during surface-mediated osteoblastic differentiation of MSCs [46]. Moreover, another non-canonical Wnt signaling protein, Wnt11, is upregulated before Wnt5a in response to the alteration in integrin heterodimers; Wnt11 leads to the production of Wnt5a and helps govern cytoskeletal polymerization and cell shape [7, 23, 38, 50].

Non-canonical signaling has been shown to be the primary regulating pathway within the family of Wnts for cells responding to microroughened Ti surfaces. Canonical Wnt signaling proteins like Wnt3a and β-catenin are upregulated during differentiation of MSCs on tissue culture polystyrene using osteogenic media containing dexamethasone and beta-glycerophosphate [13, 37]. When cells are grown on microroughened Ti substrates in growth media, Wnt3a expression is downregulated and Wnt5a is upregulated. Moreover, treatment of these cells with Wnt3a reduces production or expression of differentiation markers and osteogenic factors [2, 6].

Recent studies evaluating other Wnt signaling proteins in the context of bone fracture healing and whole bone morphology, showed that non-canonical Wnt16 was increased in the cortical bone; overexpression or deletion of Wnt16 increased or reduced cortical bone thickness, respectively, in mouse models [25, 30, 47, 48]. Both preclinical and clinical studies support the hypothesis that improved cortical bone stability is achieved when Ti implants with microroughened surfaces are used, particularly when those surfaces have a hydrophilic surface chemistry [14, 20, 56], suggesting that Wnt16 might also be involved in determining implant stability and retention via modulation of osteoblast differentiation [25, 30, 47, 48]. Therefore, the aim of this study was to evaluate the role of non-canonical Wnt16, found to be highly expressed in the cortical bone, in the process of peri-implant osteogenesis. To do this, we assessed the effect of Wnt16 on surface-mediated osteoblastic differentiation in vitro. We then examined its effect in vivo using a rat model of decreased cortical bone quality to evaluate whole bone phenotype and osseointegration.

## Materials and Methods

### Substrate Manufacturing

#### Culture Disks

Grade 2 titanium 15 mm diameter disks were punched from 1 mm thick sheets (Institut Straumann AG, Basel, Switzerland) and treated and characterized as described previously [43, 45]. Pretreatment (PT) disks were degreased, and pickle treated to obtain Ti disks with a smooth surface. Subsets of these PT disks were subsequently treated by grit-blasting and acid-etching (SLA) with large grit corundum and a combination of HCl:H_2_SO_4_. SLA disks were cleaned in HNO_3_, rinsed in deionized water, air dried, and packed in aluminum foil. A subset of SLA disks was cleaned and processed under a modified nitrogen environment and stored in saline to reduce the exposure to air (modSLA) resulting in a super hydrophilic surface. All disks were then gamma irradiated to sterilize the packaging and implant surface [4, 45].

#### Implants

Grade 4 titanium screw style implants were machined to be 2.5 mm in diameter, 3.5 mm in length, and 0.8 mm pitch (Insitut Straumann AG). Implant surfaces were prepped as described above and characterized previously [44]. modSLA treatment was used on all implants and implants were in saline for at least 1 month. Implants were then gamma irradiated to sterilize the packaging and implant surface. In this study, modSLA implants were removed from the sterile packaging in sterile conditions in a biological safety cabinet. Implants were then rinsed in ultrapure water and repackaged in the implant container (in air without saline) and aged for 1 month in an undisturbed biological safety cabinet to create hydrophobic SLAnano implants, which have comparable nanoscale topography to modSLA, but behave similarly to SLA [4, 44].

### In Vitro Assessment

#### Cell Culture

Human female bone MSCs (donor 8011L, Institute for Regenerative Medicine, Texas A&M Health Science Center, Bryan, TX, USA) were cultured in growth medium (GM) comprised of Alpha Modified Eagle Medium (αMEM, Life Technologies, Carlsbad, CA, USA), supplemented with 2 mM l-glutamine and 16.5% heat inactivated fetal bovine serum (Gemini Bioscience, Calabasas, CA, USA) at 37 °C in 5% CO_2_ and 100% humidity and cultured to 80% confluence in T75 flasks (Corning Inc., Oneonta, NY, USA) before plating on the Ti substrates. MSCs were cultured on tissue culture polystyrene (TCPS), PT, SLA, or modSLA at a density of 10,000 cells/cm^2^ at 37 °C, 5% CO_2_ and 100% humidity for all experiments. Media were changed 24 h after plating and every 48 h thereafter for 7 days. At day 7, cells were incubated with fresh media for 24 h. At harvest, conditioned media were collected from the cultures and stored at − 80 °C for biological assays.

#### Autocrine and Paracrine Effects of Wnt16

We used production of OPG as a marker of Wnt16 activity based on the observation that overexpression of Wnt16 increased OPG production in long bones of mice [16, 47]. MSCs were cultured on the Ti disks in GM; media were changed 24 h after plating and then at 48-h intervals for 7 days. Two experimental designs were used. In design 1, control cultures received vehicle and treated cultures received either 100 ng/mL or 1 μg/mL recombinant human Wnt16b (rhWnt16b) (R&D Systems, Minneapolis, MN, USA) at each media change. In design 2, cultures were grown in GM and on day 7, they were treated with GM or GM containing either 100 ng/mL or 1 μg/mL rhWnt16b. At harvest, total DNA content of the cell layers and production of OPG in the conditioned media were determined were determined as described below.

To determine if Wnt16 is produced by MSCs in response to multiscale Ti surface topography and if its production is sensitive to exogenous Wnt16, cultures were treated continuously at each media change with 100 ng/mL rhWnt16b. Cells were plated as described above, and GM and GM supplemented with exogenous rhWnt16b were changed at 24 h and then at 48-h intervals. On day 7, media were replaced with fresh GM for 24 h and the conditioned media were collected for analysis,

Autocrine/paracrine regulation by endogenous Wnt16 was examined by treating MSC cultures with 2 μg/mL rabbit polyclonal anti-human Wnt16 antibody (ThermoFisher Scientific, Waltham, MA, USA) or human IgG isotype control antibody (ThermoFisher Scientific). Cells were plated as described above, and GM and GM supplemented with blocking antibodies for Wnt16 or IgG were changed at 24 h and then at 48-h intervals. On day 7, media were replaced with fresh GM for 24 h and the conditioned media were collected.

ELISAs were used to measure levels of OCN (ThermoFisher Scientific), BMP2 (R&D Systems), OPG (R&D Systems), OPN (R&D Systems), parathyroid hormone-related peptide (PTHrP, LifeSpan Biosciences, Seattle, WA), Wnt3A (Biomatik), Wnt11 (Biomatik), and Wnt5A (Biomatik). Cell monolayers were washed twice with 0.5 mL phosphate-buffered saline (PBS), lysed in 0.05% Triton-X100, and homogenized by sonication at 40 A using a Vibra-Cell ultrasonicator (Sonics & Materials Inc., Newtown, CT, USA). DNA content in the cell layer lysate was measured with PicoGreen (Promega, Madison, WI, USA) using a Synergy H1 Hybrid Reader fluorescence detector (BioTek, Winooski, VT, USA) at an excitation of 504 nm and emission of 531 nm. Immunoassay data were normalized to DNA content.

#### Regulation of Wnt16 Production by Semaphorin 3a, Wnt11, and Wnt5a

Human female MSCs (donor 127, Ossium Health, Indianapolis, IN, USA) were cultured in GM comprised of αMEM without nucleosides, supplemented with 2 mM l-glutamine and 10% heat inactivated FBS (Gemini Bioscience) at 37 °C in 5% CO_2_ and 100% humidity and cultured to 80% confluence in T75 flasks (Corning Inc.) before plating on the surfaces. Cells were plated at a density of 5000 cells/cm^2^ at 37 °C, 5% CO_2,_ and 100% humidity for all experiments. Cultures were treated for 7 days with vehicle or soluble recombinant human semaphorin 3a (sema3a) (1 μg/mL), Wnt5a (50 ng/mL), or Wnt11 (50 ng/mL) (R&D Systems, Minneapolis, MN), based on previously defined concentrations [7, 42, 50]. 24 hours after plating, GM and supplemented GM were changed with subsequent media changes every 48 hours. Cells were incubated for 24 h on day 7 with fresh GM before harvest. At harvest, conditioned media were collected from the cultures and stored at − 80 °C, and MSCs were rinsed twice with 1x PBS and placed in 0.5 mL of Triton-X100 and stored at − 80 °C for biological assays. OCN (R&D Systems) and Wnt16 (Biomatik, Pittsburgh, PA) were measured in the conditioned media.

### Evaluation of Wnt16 on Whole Bone Morphology in a Model of Compromised Cortical Thickness

We have shown that botox-induced paralysis of the muscles overlying the femoral bone reduced cortical bone formation and increased cortical bone porosity in adult male Sprague-Dawley rats [18]. Botox also caused a loss of trabecular bone in the femoral metaphysis and reduced osseointegration of transcortical Ti implants with a hydrophilic microroughened surface. We used this model to assess the effects of Wnt16 on the impaired bone phenotype induced by botox, specifically focusing on the osseointegration of transcortical Ti implants. Rather than using hydrophilic modSLA implants, which have been shown to overcome compromised bone qualities [44], we assessed peri-implant bone formation using SLAnano implants, which have a microroughened topography with nanoscale features identical to the modSLA surface, but with a hydrophobic chemistry [45].

All animal procedures were conducted in compliance with the US National Research Council’s Guide for the Care and Use of Laboratory Animals, the US Public Health Service’s Policy on Humane Care and Use of Laboratory Animals, and Guide for the Care and Use of Laboratory Animals. Approval was obtained by Virginia Commonwealth University’s Institutional Animal Care and Use Committee (IACUC). ARRIVE guidelines and additional recommendations were used in the development, implementation, and analysis of this study. A power analysis was conducted using an alpha of 0.05 and a power of 80% (delta = 5, sigma = 3, *m* = 1) to reveal a minimum of *n* = 7 per group for the study to yield statistical significance. Extra rats were used to ensure that experimental exclusions did not reduce power below the threshold.

#### Effect of rhWnt16b on Bone Phenotype

Botulinum toxin type A (onabotulinumtoxin A; BOTOX^®^, Allergan, Inc. an AbbVie, Inc. company, Lake Bluff, IL, USA; [botox]) was dissolved in 0.9% saline (10 units/mL) and used to decrease bone quality and implant osseointegration, as described previously [18]. Study design included 4 groups: vehicle (*n* = 4), botox (*n* = 8), vehicle + rhWnt16b (*n* = 9), and botox + rhWnt16b (*n* = 9). Male Sprague-Dawley (SD) rats that were between 300 and 325 grams were purchased (Charles River Laboratories, Wilmington, MA, USA) and acclimated at VCU under single housing, access to food and water ad libitum. On day 1 and day 28 of the study, SD rats were anesthetized by 5% isoflurane gas inhalation and maintained and treated based on the experimental groups at 2.5% isoflurane. Botox designated SD rats were injected with 2 units of botox into each of the paraspinal, quadricep, hamstring, and calf muscles in the right hindlimb for a total of 8 units of botox in the right hindlimb. Rats were allowed to recover on a heated blanket and then returned to their cage for 3 weeks. On day 33 and day 39, SD rats were anesthetized by 5% isoflurane gas inhalation and maintained at 2.5% isoflurane during treatment. Treatment consisted of either 20 μL vehicle (1× PBS) or 20 μL vehicle containing 2 μL rhWnt16b (R&D Systems, 100 μg/mL) injected into the periosteal layer of the midshaft of the right femur. Contralateral legs received a 1× PBS injection. Control rats received 1× PBS in each femur to reduce the necessary number of rats needed. On day 49, rats were humanely euthanized by CO_2_ inhalation and cervical dislocation. Both femurs were harvested, the treatment leg and the control (not treated), and unfrozen fresh wet femurs were evaluated by micro-CT analysis and 3-point bending fracture analysis. Data are normalized as treatment over contralateral leg (right leg is treatment/left leg is contralateral).

#### Effect of rhWnt16b on Osseointegration

Four groups were used to evaluate implant osseointegration using SLAnano implants. Groups were control, botox, vehicle + rhWnt16b, and botox + rhWnt16b. Male Sprague-Dawley rats that were between 300 and 325 g were purchased (Charles River Laboratories) and acclimated at VCU under single housing, access to food and water ad libitum. A schematic of the study design is shown in Supplemental Fig. 1. On day 1 and day 28 of the study, SD rats were anesthetized by 5% isoflurane gas inhalation and maintained and treated based on the experimental groups at 2.5% isoflurane. Botox designated SD rats were injected with 2 units of botox into each of the paraspinal, quadricep, hamstring, and calf muscles in the right hindlimb for a total of 8 units of botox (Allergan, Inc.) in the right hindlimb. Rats were allowed to recover on a heated blanket and then returned to their cage for 3 weeks.

**Fig. 1:**
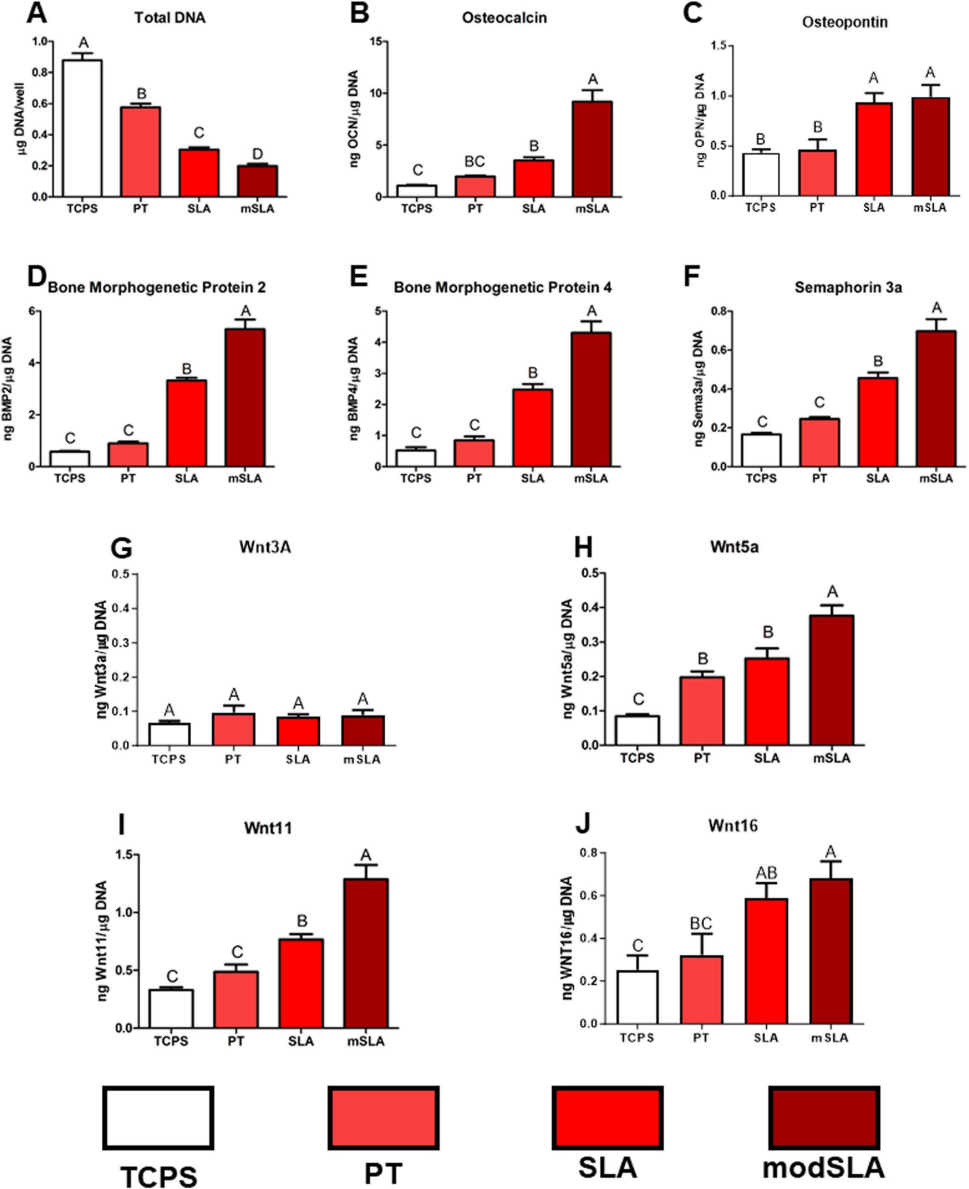
Surface-dependent Wnt production by human bone MSCs. MSCs were cultured for 7 days on Ti disks possessing microroughness and increased wettability. **A** Total DNA content was determined in the cell layer lysate. **B** Osteocalcin (OCN), **C** osteopontin (OPN), **D** bone morphogenetic protein 2 (BMP2), **E** BMP4, **F** sema3a, **G** canonical Wnt3a, **H** non-canonical Wnt5a, **I** Wnt11, and **J** Wnt16 were determined in the conditioned media after 24 h. Letters on each bar indicate significant differences among groups. Groups that do not share the same letters are significant at *p* < 0.05; *n* = 6 per culture/variable. *TCPS* tissue culture polystyrene, *PT* pretreated Ti, *SLA* large grit blasted/acid-etched microroughened surface, *modSLA* hydrophilic SLA

On day 21, SD rats were anesthetized by 5% isoflurane gas inhalation and maintained at 2.5% isoflurane. The hindlimbs were prepared by shaving and cleaning using ethanol and chlorhexidine. The starting surgical leg was chosen at random. An 8 mm incision was made over the medial side of the knee and the distal metaphysis was exposed by blunt dissection and the periosteum was removed. A dental handpiece was used to create a 2.2 mm diameter and 3.5 mm in depth. Titanium implants (SLAnano) were placed unicortical into the distal metaphysis of each femur till flush with the cortical surface and hand-tightened using a custom-made implant driver. Titanium cover screws were placed on the end of the implant to prevent bone growth over-top of the implant. The periosteum and muscle were re-apposed and sutured in place using resorbable sutures, and the skin was closed with 9 mm wound clips. The contralateral leg then underwent the same surgical procedure. SD rats recovered from anesthesia on a water-circulating warming pad and injected subcutaneously with 1 mg/kg buprenorphine SR LAB (ZooPharm/Wildlife Pharmaceuticals, Inc., Laramie, WY, USA).

We inserted an implant in the contralateral leg as a control, enabling us to study the effect of the Wnt16b treatment on implant osseointegration in the same animal. The presence of the implant does not disturb the animal’s function. Moreover, by placing an implant in each limb, we eliminated the variability of altered weight bearing between the treatment and control limbs.

On day 33 and day 39, following recovery from implant surgery and separation from the second botox injection, SD rats were anesthetized by 5% isoflurane gas inhalation and maintained at 2.5% isoflurane. Treatment consisted of either 20 μL vehicle (1× PBS) or 20 μL containing 2 μL Wnt16 (R&D Systems, 100 μg/mL) injected into the periosteal layer directly adjacent to the implant of the right femur. The contralateral leg received a 1× PBS injection. The control group received 1× PBS in each femur to reduce the number of rats needed. On day 49, rats were humanely euthanized by CO_2_ inhalation and cervical dislocation. Both femurs were harvested, and fresh wet femurs were evaluated by micro-CT analysis and mounted in mechanical testing clamps with quick setting epoxy overnight and then subsequently tested by mechanical torsion to failure.

#### Micro Computed Tomography Analysis

Micro-computed tomography (micro-CT) (SkyScan 1173, Bruker, Kontich, Belgium) was used to evaluate the bone phenotype, as well as peri-implant growth. Femurs were isolated and stored on ice at 4 °C and scanned fresh without fixation within 24 h of harvest to conduct mechanical torque testing on the same samples.

Both distal and proximal ends of the femurs were scanned at a resolution of 1120 × 1120 pixels (isotropic voxel size of 15.82 μm) using a 0.25 mm brass filter, at an exposure of 420 ms, with scanning energies of 120 kV and 66 μA. Five X-ray projections were acquired every 0.2° and averaged. A standard Feldkamp reconstruction was performed by NRecon Software (Bruker) with a beam hardening correction of 20%, and no smoothing was applied. Quantitative trabecular bone morphometric parameters, including bone volume/total volume (BV/TV), trabecular number, trabecular thickness, and total porosity, were determined. Quantitative cortical bone morphometric parameters, such as BV/TV, total porosity, mean total cross-sectional bone area, mean cross-sectional bone perimeter, and cortical thickness were determined.

Bone-to-implant contact (BIC) was evaluated for each implant by scanning at the distal femoral metaphysis at a resolution of 1120 × 1120 pixels (isotropic voxel size of 15.8 μm) using 0.25 mm brass filter and at an exposure of 420 ms, 120 kV voltage and 66 μA current. After reconstruction using the same method as described above, a volume of interest (VOI) was selected to analyze BIC at different conditions. For total BIC, the VOI was selected from the bottom of the implant toward the apex to the highest position that cortical bone was still in contact with the implant surface. For bone marrow-to-implant contact, the VOI was selected in the bone marrow region. For cortical BIC, the VOI was selected in the region where implant surfaces were in contact with cortical bone. The VOI was shrink-wrapped, dilated 2 pixels around the implant, and subtracted from the original VOI, which enabled us to remove the implant from the calculation. The implant VOI was loaded as the region of interest. The new VOI was dilated 2 pixels away from the implant VOI and subtracted from the implant VOI. The bone volume within the new VOI was calculated and normalized to the new VOI, which was recorded as BIC.

#### Mechanical Evaluation

Mechanical torque to failure was used to determine the overall implant mechanical integrity using a Bose ElectroForce 3200 Series III Axial-Torsion mechanical testing system equipped with a 445 N/5.7 N-m load/torque transducer. The load cell was zeroed, and a 0.1 Hz filter was applied to reduce noise.

For 3-point bending analysis, bones were placed so that the injection site of the midshaft of the femur is centered on two support struts facing upward (Bose). The load cell was mounted with a triangular prism pointed testing mount and axial displacement was achieved by axial compressive displacement at a rate of 0.1 mm/s till failure [17, 19].

In the second experiment, torsional testing was conducted on the implants. Implants were first attached to a custom-made implant mount, using one stainless-steel implant mount welded into custom machined block with thread holes that match the fixation mounts on the mechanical testing device. Once the implant was secured tightly to the mount and was perpendicular to the axis of rotation, the sides of the cortical bone were then secured between two flat specimen clamps, ensuring that significant preload induction (> ± 0.15 N load) or torque (< ± 0.1 N cm) would not occur. The femurs were then rotated at 0.1° s^−1^ to remove the implant from the surrounding bone. At the same time, the mechanical testing frame raised the mount at a rate of 0.8 mm/360° to ensure the threads of the mount would not catch on the implant threaded grooves and create compressive loads. Data collection was achieved at 100 Hz to 60° of rotation.

Mechanical evaluation was determined by generating load vs displacement for 3-point bending analyses and torque vs degree graphs for torsional analyses after the load and displacement or torque and rotation were all normalized to the same starting conditions (0 N cm and 0°). Curves were fit to a bilinear model to distinguish the linear region from the toe region (SLM-Shape Language Modeling version 1.14, MATLAB, MathWorks, Natick, MA, USA) and were evaluated for the maximum load at failure (N), stiffness (N m), and energy at failure (millijoules) and normalized to cross-sectional area calculated in the micro-CT analysis for each leg 3-point bending analyses. Torque at failure (N m), torsional stiffness (linear region slope, N m/radians), and torsional energy (area under the curve, millijoules) were calculated from the torque vs degree graphs for the implant osseointegration analysis. Data are represented as treatment (right leg) over control (left leg).

### Statistical Analysis

Data are means ± standard error mean of six independent cultures/variable for in vitro experiments. In vitro cell experiments were repeated to ensure the validity of the data. Experiments with more than two groups were subjected to a two–way analysis of variance with a two-tailed Tukey correction to adjust for multiple comparisons to maintain an experiment-wise error rate (*α*) of 0.05 using JMP statistical software (SAS Institute Inc., Cary, North Carolina). In vivo assessment was done between contralateral legs and treatment legs by Wilcoxon matched pairs signed rank test (*α* = 0.05) represented by asterisk (*) and a one-way analysis of variance to compare between groups using GraphPad Prism v5.01 (GraphPad, La Jolla, CA, USA).

## Results

### Wnt16 Production was Sensitive to Surface Topography

Compared to cultures grown on TCPS, MSCs exhibited reduced total DNA content on all Ti surfaces, and this was greatest on modSLA (Fig. 1A). OCN was increased on SLA and modSLA compared to TCPS and highest production was seen on modSLA (Fig. 1B). OPN was increased on both SLA and modSLA to similar levels versus TCPS (Fig. 1C). Local factors BMP2, BMP4, and sema3a were increased on SLA and further increased on modSLA (Fig. 1D–F). Canonical Wnt3a was not changed across surfaces, while non-canonical Wnt5a and Wnt11 were highest on modSLA, and SLA was increased compared to PT with Wnt11 on Wnt16 production. (Fig. 1G–I). Wnt16 was increased on SLA and modSLA compared to TCPS at 7 days, and PT was not different from SLA or TCPS on Wnt16 production (Fig. 1J).

### Wnt16 Regulates MSCs in a Surface-Dependent Manner and Reduces the Impact of Surface Microroughness on Osteoblast Differentiation

To select the time and dose of rhWnt16b to use on the MSCs grown on the different implant surfaces, we examined the effect of different doses of rhWnt16b on DNA and OPG. The effect of exogenous rhWnt16b varied with the treatment regimen. DNA content of the cultures grown on modSLA was increased compared to TCPS only when treated continuously and with the lower concentration (Supplemental Fig. 2a). MSC cultures grown on modSLA exhibited increased production of OPG compared to TCPS, but only cells treated continuously with 100 ng/mL rhWnt16b exhibited a further increase in OPG production (Supplemental Fig. 1b). Based on these data, further studies used the continuous treatment protocol and 100 ng/mL rhWnt16b.

**Fig. 2:**
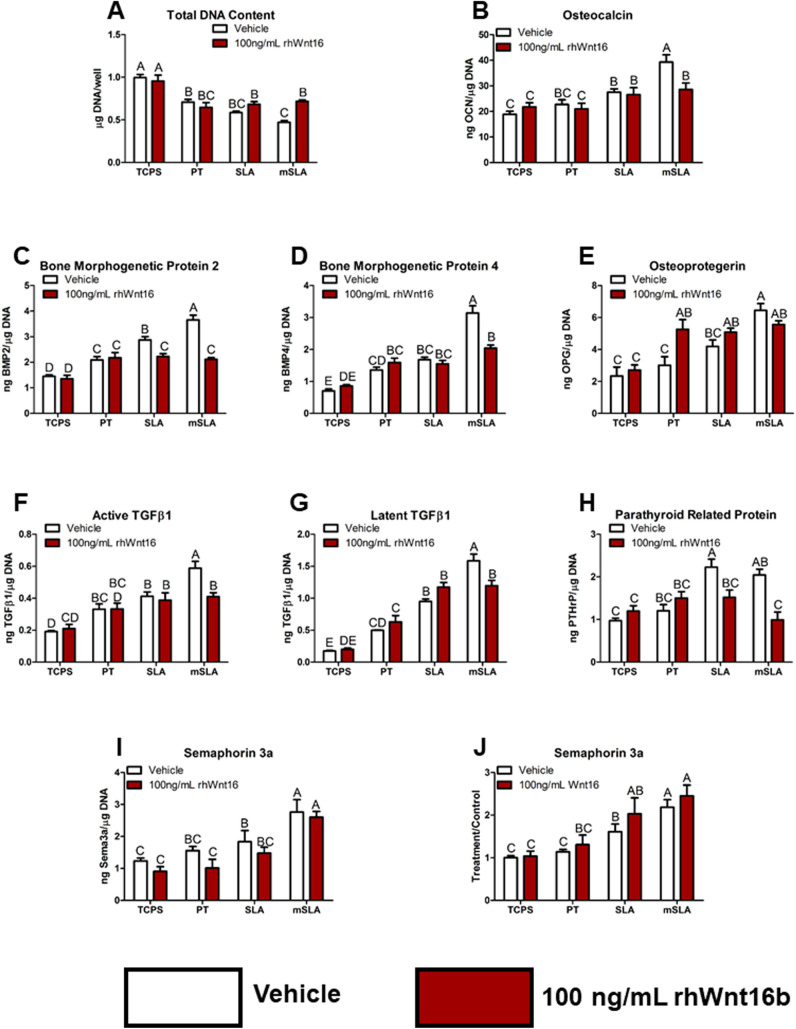
Bone MSC response at 7 days of culture on Ti implant surfaces possessing microroughness and increased wettability following continuous treatment with 100 ng/mL rhWnt16b. **A** Total DNA content was determined in the cell layer lysate. **B** Osteocalcin, **C** bone morphogenetic protein 2 and **D** BMP4, **E** osteoprotegerin, **F** active and **G** latent transforming growth factor beta 1, **H** parathyroid hormone-related peptide, and **I** semaphorin 3a were determined in the conditioned media after 24 h. **J** Treatment over control ratio of two donors and four experiments for semaphorin 3a. Letters on each bar indicate significant differences among groups. Groups that do not share the same letters are significant at *p* < 0.05; *n* = 6 per culture/variable. *TCPS* tissue culture polystyrene, *PT* pretreated Ti, *SLA* large grit, acid-etched microroughened implant, *modSLA* hydrophilic SLA

The effect of exogenous rhWnt16b was surface dependent. Total DNA content was decreased on all Ti surfaces compared to TCPS, with the greatest decrease on modSLA (Fig. 2A). Treatment with rhWnt16b restored DNA content in the modSLA cultures to that of the other Ti substrates. OCN was increased on SLA and modSLA compared to TCPS; this effect was blocked by rhWnt16b treatment on modSLA (Fig. 2B). BMP2 was increased in a surface roughness dependent manner with the highest increase observed in the modSLA cultures; treatment with rhWnt16b reduced production on SLA and modSLA to levels observed in the PT cultures (Fig. 2C). Similarly, BMP4 production was elevated on the Ti substrates, with the highest levels on modSLA; rhWnt16b did not alter the surface effect on PT or SLA, but it blocked the further increase on modSLA (Fig. 2D). In contrast, rhWnt16b stimulated OPG production on the PT surface to levels observed on the SLA and modSLA substrates (Fig. 2E). Active and latent TGFβ1 were increased on SLA and further increased on modSLA; rhWnt16b decreased production on modSLA to levels observed on SLA (Fig. 2F, G). PTHrP production on SLA and modSLA was reduced by rhWnt16b treatment to levels observed on TCPS (Fig. 2H). Lastly, sema3a exhibited a surface-dependent increase in production; rhWnt16b had no effect on sema3a on any of the surfaces (Fig. 2I) and this was confirmed comparing treatment/control ratios 4 separate experiments (Fig. 2J).

### Inhibition of Endogenous Wnt16 Activity Reduced DNA Content and Increased Osteoblast Differentiation and Production of Osteogenic Factors

**Fig. 3:**
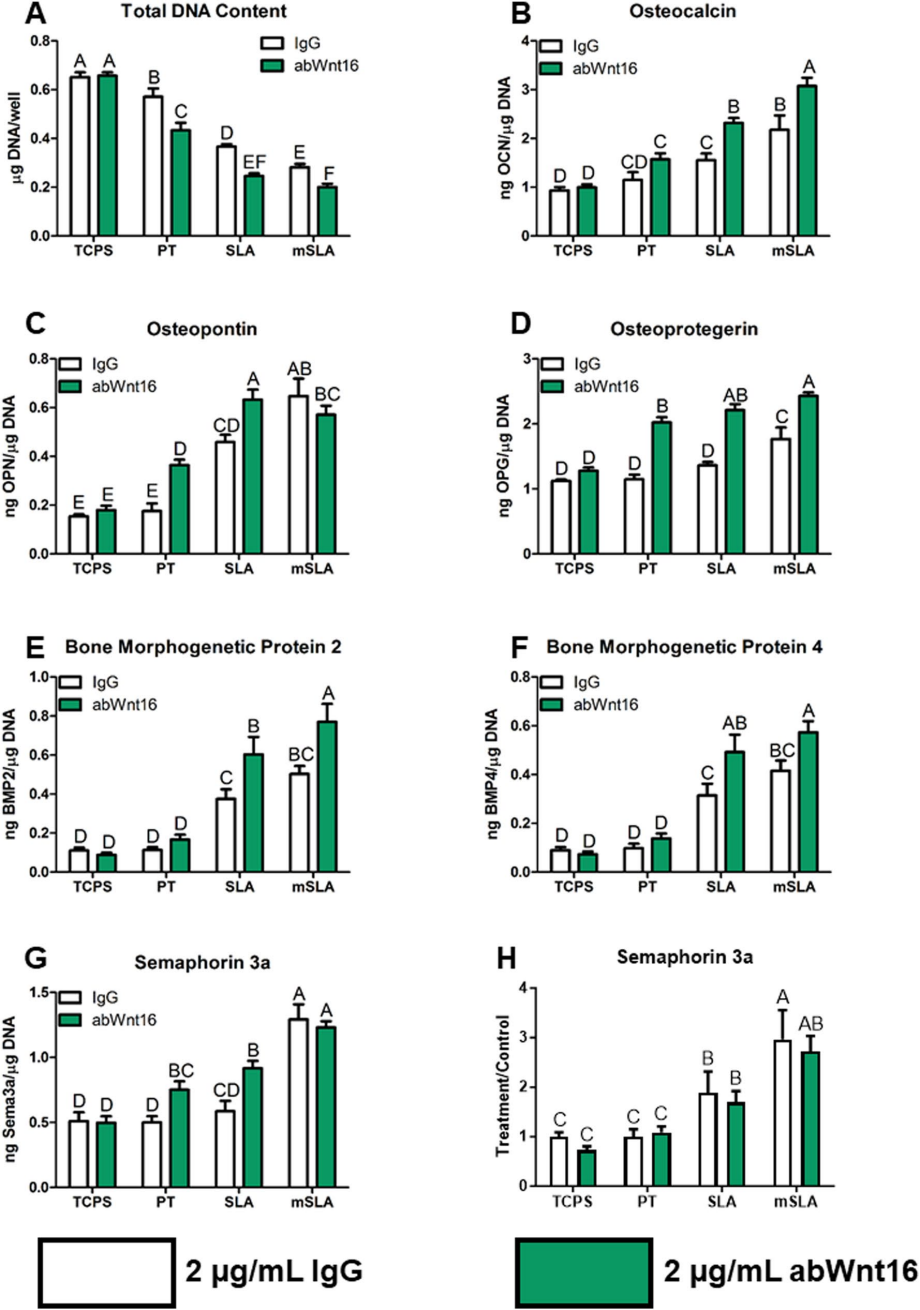
Bone MSC response at 7 days of culture on Ti implant surfaces possessing microroughness and increased wettability with treatment of 2 μg/mL abWnt16 continuously. **A** Total DNA content was determined in the cell layer lysate. **B** osteocalcin, **C** osteopontin, **D** osteoprotegerin, **E** bone morphogenetic protein 2 and **F** BMP4, and **G** semaphorin 3a were determined in the conditioned media after 24 h. **H** Treatment over control of two donors and three experiments for semaphorin 3a. Letters on each bar indicate significant differences among groups. Groups that do not share the same letters are significant at *p* < 0.05; *n* = 6 per culture/variable. *TCPS* tissue culture polystyrene, *PT* pretreated Ti, *SLA* large grit, acid-etched microroughened implant, *modSLA* hydrophilic SLA

Blocking endogenous Wnt16 activity by treating the cultures with anti-Wnt16 polyclonal antibody resulted in a decrease in total DNA content on PT, SLA, and modSLA (Fig. 3A). OCN was increased on SLA and modSLA (Fig. 3B), while OPN was increased on SLA and PT but not on modSLA (Fig. 3C). OPG was increased by anti-Wnt16 antibody treatment on all Ti surfaces (Fig. 3D). BMP2 and BMP4 were increased on SLA and modSLA (Fig. 3E, F). Lastly, sema3a was increased by antibody treatment only on PT and SLA substrates (Fig. 3G). However, this effect was lost when assessing the effects of blocking antibody treatment for both donors and three independent experiments (Fig. 3H).

### Production of Wnt16 was Regulated in a Surface-Dependent Manner

**Fig. 4:**
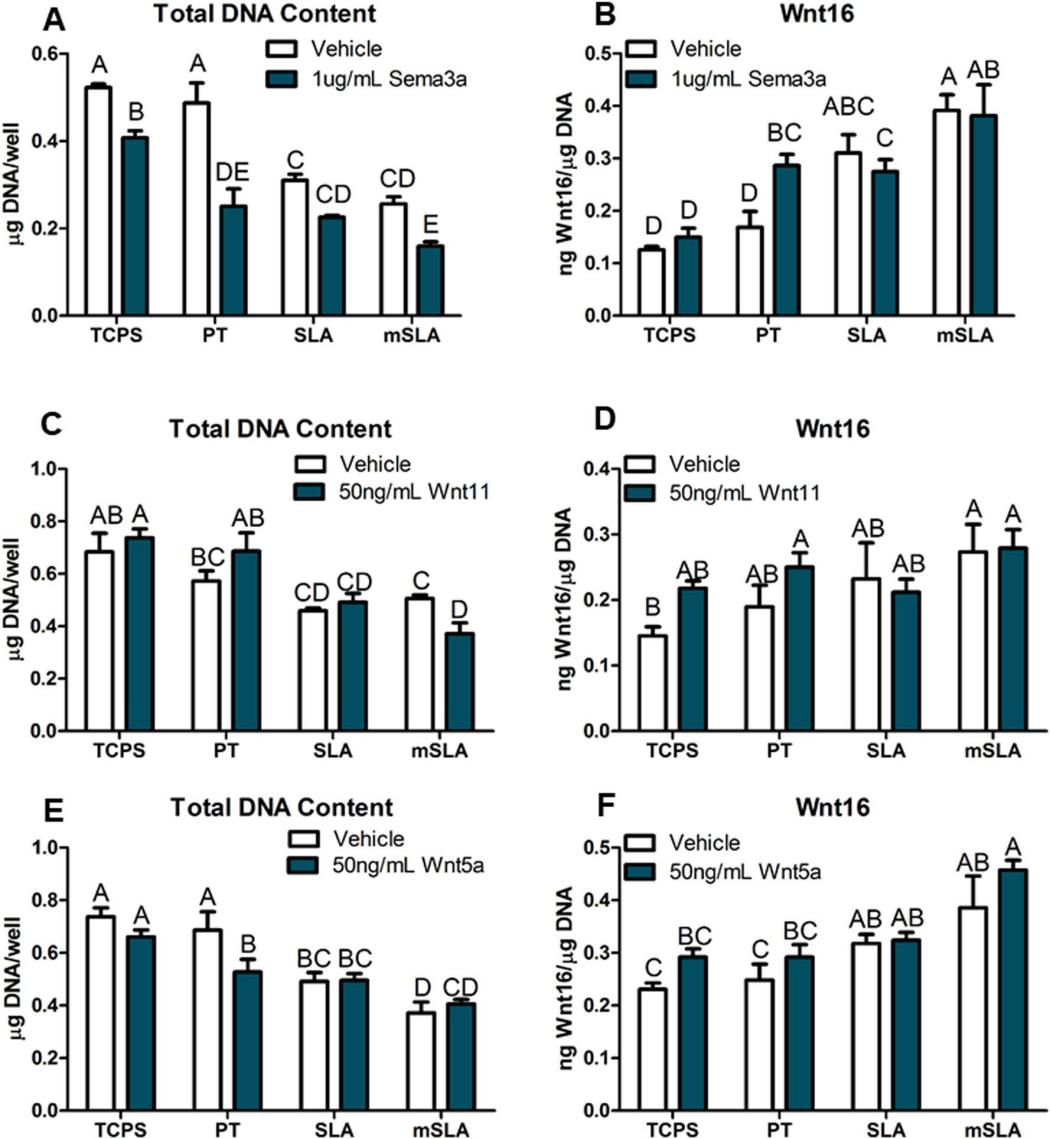
Effect of non-canonical Wnts and Semaphorin 3a on Wnt16 production by bone MSCs during surface-mediated differentiation. **A** Total DNA content and **B** Wnt16 after treatment with 1 μg/mL sema3a for 7 days. **C** Total DNA content and **D** Wnt16 after treatment with 50 ng/mL Wnt11 for 7 days. **E** Total DNA content and **F** Wnt16 after treatment with 50 ng/mL Wnt5a for 7 days. Letters on each bar indicate significant differences among groups. Groups that do not share the same letters are significant at *p* < 0.05; *n* = 6 per culture/variable. *TCPS* tissue culture polystyrene, *PT* pretreated Ti, *SLA* large grit, acid-etched microroughened implant, *modSLA* hydrophilic SLA

Treatment with sema3a decreased total DNA content of cultures on TCPS, PT, and modSLA surfaces (Fig. 4A), but only increased Wnt16 on the PT surface over the surface-dependent increase (Fig. 4B). Wnt11 treatment decreased DNA content of cultures grown on modSLA (Fig. 4C). Wnt16 production was increased by cells on modSLA compared to TCPS, but Wnt11 had no effect on Wnt16 production on any of the surfaces (Fig. 4D). Wnt5a treatment did not affect total DNA content on SLA or modSLA but reduced content on PT surfaces (Fig. 4E). It had no effect on Wnt16 production on any surface (Fig. 4F).

### Wnt16 Did Not Restore the Bone Phenotype Induced by Botox

**Fig. 5:**
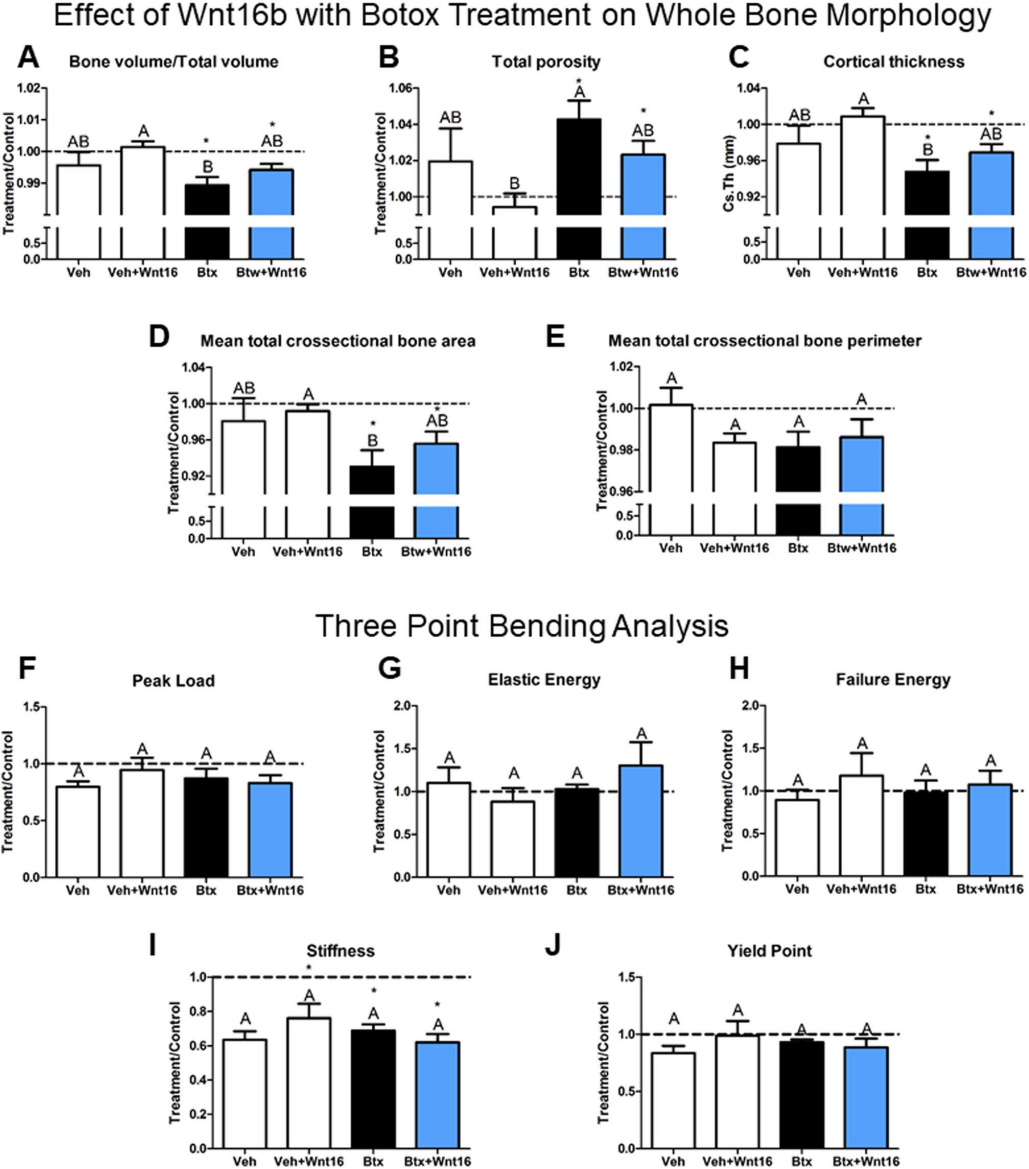
Effect of rhWnt16b in a botox-induced model of impaired cortical bone properties. **A** Bone volume/total volume, **B** total porosity, **C** cortical thickness, **D** mean cross-sectional bone area, and **E** total cross-sectional bone perimeter were determined around the injection site (2 μg rhWnt16b) by micro-CT. Three-point bending analysis of femurs was used to quantify **F** peak load, **G** elastic energy, **H** failure energy, **I** stiffness, and **J** yield point after bilinear curve fitting. Data are treatment over control (right leg = treatment leg, left leg contralateral). Letters on each bar indicate significant differences among groups. Groups that do not share the same letters are significant at *p* < 0.05; *n* = 6 per culture/variable. *Statistical difference from the contralateral leg at a *p* < 0.05. Veh—vehicle (*n* = 4), Veh + rhWnt16b—vehicle plus twice 2 μg rhWnt16b injections (*n* = 8), Btx—botox injections to impair cortical bone (*n* = 9), Btx + rhWnt16b—botox-injected rats treated with twice 2 μg rhWnt16b injections (*n* = 9)

rhWnt16b did not affect BV/TV, total porosity, cortical thickness, cross-sectional bone area, or perimeter in either botox-treated bone or in the healthy control bones (Fig. 5A–E). Botox treatment reduced BV/TV compared to the contralateral leg. rhWnt16b treatment of botox-injected hindlimbs did not improve BV/TV nor did rhWnt16b enhance BV/TV in the control femurs (Fig. 5A). Botox treatment increased total porosity compared to the contralateral leg and rhWnt16b treatment of botox-injected hindlimbs did not decrease porosity compared to the contralateral hindlimb (Fig. 5B). Both cortical thickness and cross-sectional bone area were diminished with botox treatment and were not improved in the botox + Wnt16-treated hindlimbs (Fig. 5C, D). No differences were seen in the cross-sectional bone perimeter (Fig. 5E).

Mechanical analysis of these hindlimbs by three-point bending showed no differences between vehicle and botox treatment with or without rhWnt16b treatment for peak load, elastic energy, and failure energy (Fig. 5F–H). However, the stiffness was decreased compared to the contralateral leg in vehicle + rhWnt16b, botox, and botox + rhWnt16b groups (Fig. 5I). Yield point was not altered (Fig. 5J).

### Wnt16 Enhanced Osseointegration in Botox-Treated Femurs but Reduced the Quality of the Peri-Implant Bone

**Fig. 6:**
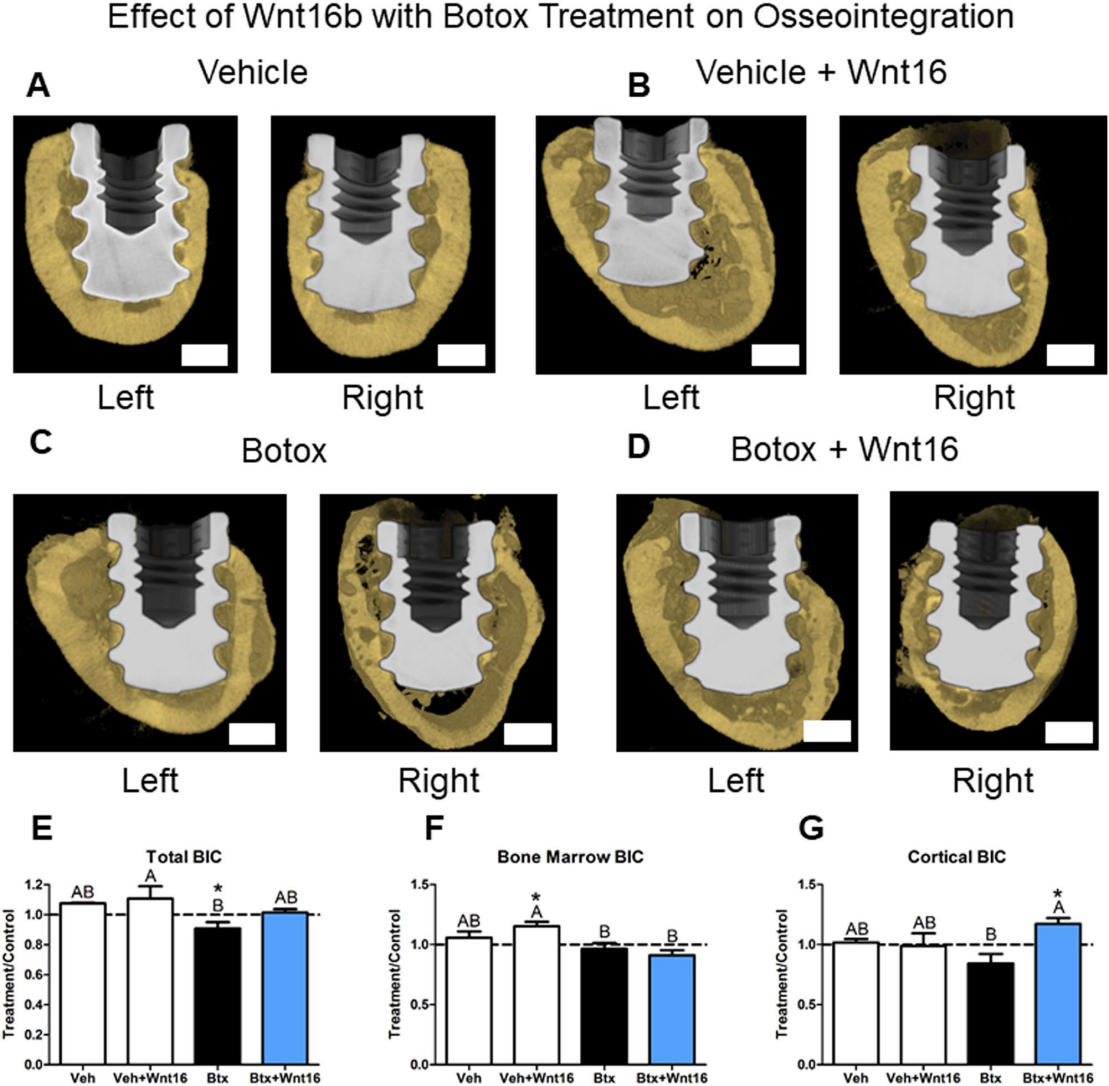
Effect of rhWnt16b (Wnt16) on osseointegration in a botox-induced model of impaired cortical bone properties. Representative images of **A** vehicle implants, **B** vehicle + Wnt16-treated implants (left control, right treatment), **C** botox implants (left control, right treatment), **D** botox + Wnt16-treated implants after 28 days of integration assessed by micro-CT. (Left control, right treatment), **E** total BIC, **F** bone marrow BIC, and **G** cortical BIC were quantified using the micro-CT reconstructions. Data are treatment over control ratios (right leg = treatment leg, left leg contralateral). Letters on each bar indicate significant differences among groups. Groups that do not share the same letters are significant at *p* < 0.05; *n* = 6 per culture/variable. *Statistical difference from the contralateral leg at a *p* < 0.05. Veh—vehicle (*n* = 4), Veh + rhWnt16b—vehicle plus twice 2 μg rhWnt16b injections (*n* = 8), Btx—botox injections to impair cortical bone (*n* = 9), Btx + rhWnt16b—botox-injected rats treated with twice 2 μg rhWnt16b injections (*n* = 9)

Osseointegration was evaluated using hydrophobic microroughened SLAnano implants. Representative micro-CT reconstructs of the implants are shown in Fig. 6 for sites treated with: vehicle (Fig. 6A), vehicle + rhWnt16b (Fig. 6B), botox (Fig. 6C), and botox + rhWnt16b (Fig. 6D). There was a decrease in cortical bone thickness and increase in cortical porosity with botox treatment. Quantitative analysis of BIC showed that botox reduced total BIC compared to the contralateral leg (Fig. 6E). Interestingly, rhWnt16b treatment of botox-treated hindlimbs was not different from the contralateral leg and vehicle or vehicle + rhWnt16b-treated groups (Fig. 6E). Further evaluation of the bone marrow space showed that BIC was increased compared to contralateral legs in vehicle + rhWnt16b-treated rats only (Fig. 6F) and was different from both botox and botox + rhWnt16b rats. Cortical bone-to-implant was unaltered in the vehicle-treated rats and while BIC was increased compared to contralateral in the botox + rhWnt16b-treated group (Fig. 6G) and was different from botox.

**Fig. 7:**
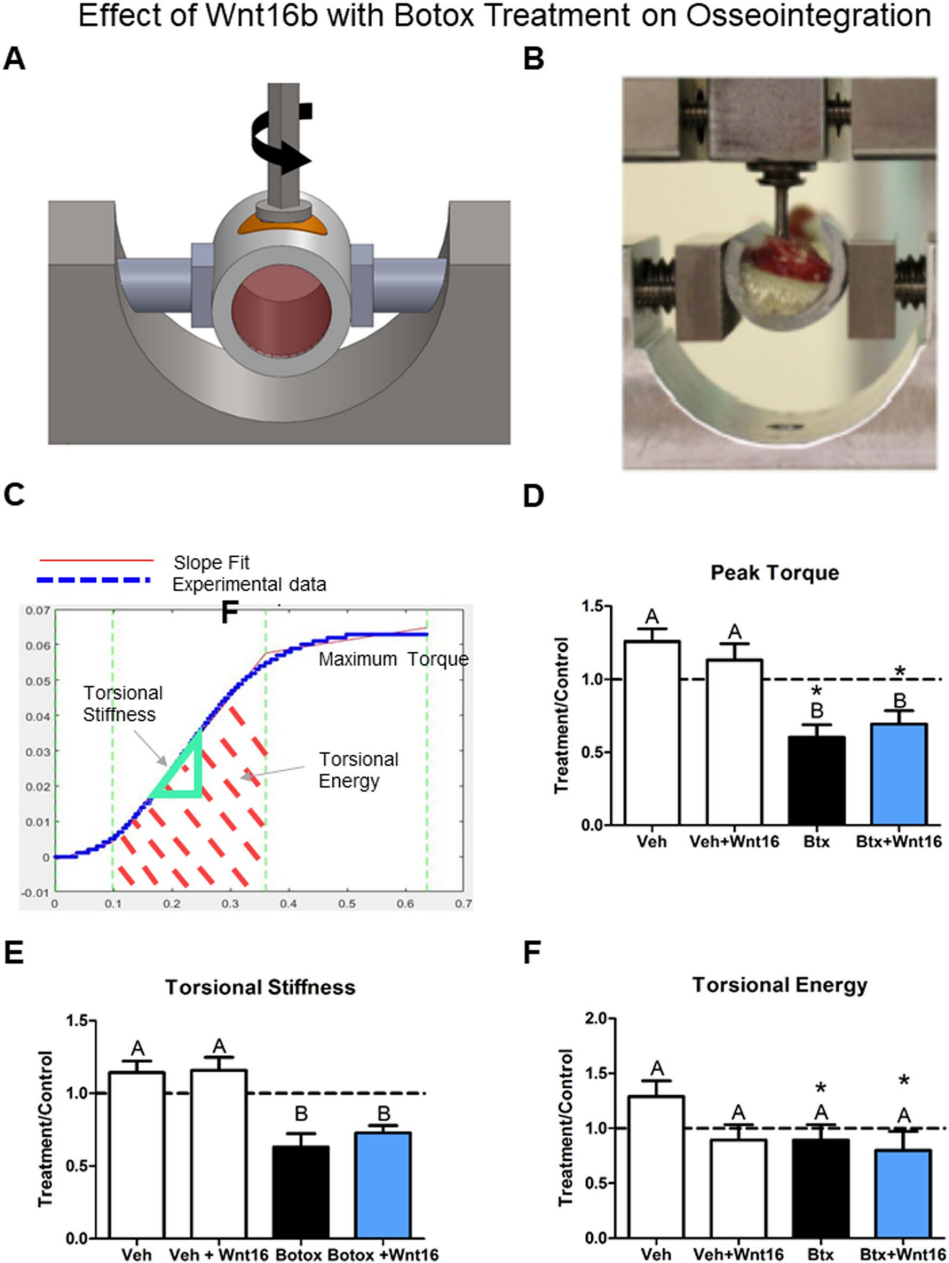
Effect of rhWnt16b (Wnt16) on mechanical anchorage during osseointegration in a botox-induced model of impaired cortical bone properties. **A** Representative torsion vs rotation curve generated in MATLAB for each implant. Quantification of **B** peak torque, **C** torsional stiffness, and **D** torsional energy for each implant group. Data are treatment over control ratios (right leg = treatment leg, left leg contralateral). Letters on each bar indicate significant differences among groups. Groups that do not share the same letters are significant at *p* < 0.05; *n* = 6 per culture/variable. *Statistical difference from the contralateral leg at a *p* < 0.05. Veh—vehicle (*n* = 4), Veh + rhWnt16b—vehicle plus twice 2 μg rhWnt16b injections (*n* = 8), Btx—botox injections to impair cortical bone (*n* = 9), Btx + rhWnt16b—botox-injected rats treated with twice 2 μg rhWnt16b injections (*n* = 9)

Mechanical torque to failure was used to further quantify the effect of botox and rhWnt16b treatment on osseointegration. A schematic of the instrument use to measure the removal torque of the implants is shown in Fig. 7A. The apparatus used to measure the removal torque of the implants is shown in Fig. 7B. First, a MATLAB graphic showing a torque vs degree plot containing the measurement parameters was generated (Fig. 7C). Analysis of peak torque showed a robust effect of botox treatment, reducing peak torque significantly compared to vehicle or vehicle + rhWnt16b. Additionally, both botox and botox + Wnt16 rats treatment limbs’ peak torque was decreased compared to their contralateral limbs (Fig. 7D). Torsional stiffness was similar to peak torque; however, no treatment limbs were different from contralateral limbs, and botox reduced stiffness in both groups receiving botox (Fig. 7E). Lastly, torsional energy absorbed till failure was not altered between groups. Botox and botox + Wnt16 hindlimbs’ torsional energy was decreased compared to contralateral limbs (Fig. 7F).

## Discussion

This study demonstrates a complex regulatory function of Wnt16 during surface-mediated osteoblast differentiation and production of factors involved in peri-implant osteogenesis. As we have reported previously, the MSCs exhibited increases in osteoblastic differentiation markers such as OCN and OPN when grown on Ti substrates with biomimetic surface topography that are correlated with increased bone formation clinically [14, 56]. In addition, MSCs produced an orchestrated cascade of local factors when responding to the biomimetic implant surface properties [9, 42, 50]. BMP2, BMP4, and sema3a stimulate osteoblast differentiation; TGFβ1 stimulates extracellular matrix production; VEGF promotes vasculogenesis; and OPG and class 3 semaphorins (3A,3C,3E) mediate the rate and extent of osteoclast activity [54, 55]. As a result, net new bone formation is achieved. The results of the present study suggest that MSCs also produce factors like Wnt16 that do not increase osteoblast differentiation, yet contribute to the ultimate osseointegration of microtextured Ti implants.

Wnt signaling proteins play an important role in peri-implant bone formation. Surface-mediated differentiation of MSCs into osteoblasts occurs in conventional MSC growth media and relies on non-canonical and calcium-dependent Wnt signaling proteins like Wnt11 and Wnt5a. In addition, the levels of β-catenin-dependent Wnts like Wnt3a are reduced in cultures grown on these surfaces; treatment with Wnt3a stimulates proliferation but downregulates differentiation [6, 42]. In contrast, Wnt3a plays a major role when MSCs are cultured on TCPS in osteogenic media [32], indicating that different mechanisms are involved.

The current study was undertaken to test the hypothesis that non-canonical Wnt16 would also promote osteoblast differentiation. Like the other non-canonical Wnts examined thus far, its production was increased on Ti substrates with biomimetic micro/nanotopography and hydrophilic surface chemistry. Surprisingly, it did not promote differentiation on these surfaces. Key local factors like BMP2, BMP4, active TGFβ1, and PTHrP were all inhibited with exogenous rhWnt16b treatment. Moreover, treatment with anti-Wnt16 antibody prevented endogenous activity of rhWnt16b and resulted in effects that were opposite to those elicited by exogenous supplementation.

These observations suggest that Wnt16 acts as a break on differentiation of osteoblasts induced by Wnt5a and downstream production of BMP2, thereby enabling proliferation of osteoprogenitor cells and increasing the pool of cells needed for net new bone formation [50, 52]. The increase in DNA content in response to rhWnt16b and the reduction in DNA content observed in cultures treated with anti-Wnt16 antibody support this hypothesis. Production of sema3a, which stimulates osteoblast differentiation, was not affected by rhWnt16b, suggesting that it may act as a compensatory mechanism when local factor production is inhibited by the proliferative signaling of Wnt16 [29, 42, 59, 63]. Sema3a regulates Wnt16 production as demonstrated by the stimulation of Wnt16 in cells cultured on the PT surface to levels observed on the microtextured SLA and modSLA surfaces. In contrast, neither Wnt11 nor Wnt5a, which stimulate BMP2 production, had a regulatory effect on Wnt16.

Others have reported that Wnt16’s primary method of action is by the upregulation of OPG in early-stage progenitor cells [48]. In addition, Wnt16 produced by mouse and human osteoblasts can act directly on osteoclasts to reduce their activity [47]. In the present study, OPG production was increased on modSLA and rhWnt16b stimulated production on the PT surface to levels observed on the microtextured substrates, indicating that maximal upregulation was achieved via cytoskeletal and mechanosensitive signals due to the surface microtexture [36]. The data also suggest that elevated levels of endogenous Wnt16 on these substrates may play a role since exogenous rhWnt16b did not cause a further stimulatory effect on OPG than was due to micro/nanotopography and hydrophilicity [55]. OPG is a decoy receptor for RANKL, preventing RANKL from binding to RANK on osteoclast progenitor cells and promoting their differentiation [11, 35, 43]. MSCs produce other factors that reduce osteoclast activity when grown on Ti surfaces with a biomimetic micro/nanotopography and hydrophilic chemistry, such as sema3c [18], suggesting that in addition to its role in supporting osteoprogenitor cell proliferation, Wnt16 plays a role in modulating the rate and extent of osteoclastic resorption [25, 30, 47, 48].

Wnt16 expression and production were not regulated by Wnt11 or Wnt5a. One possibility is that it is upstream of these factors. Alternatively, it is regulated by a different mechanism, such as via sema3a, and acts independently, likely on cell-cycle modulators [25, 30]. Wnt16 can signal via β-catenin-dependent mechanisms, similar to Wnt3a but to a lesser extent [47, 48]. This may contribute to its role in maintaining proliferative capacity of the MSCs even after Wnt3a is downregulated by Wnt5a and differentiation is stimulated on the microtextured substrates.

Others have used Wnt16 knock-in overexpression in mice to get a better understanding of its role in bone homeostasis and have shown that there was a net increase in cortical bone volume and thickness through OPG-related pathways [47, 48]. These observations indicated that Wnt16 contributed to maintenance of a healthy bone phenotype and suggested that rhWnt16b might be a useful therapeutic for improving peri-implant bone formation in a model of compromised bone quality. To test this hypothesis, we used a model of impaired bone quality created by mechanical unloading of the rat femur resulting from intramuscular botox injections. rhWnt16b delivered to the femoral bone via injection into the periosteum did not alter cortical bone quality in normal rats or in the botox-treated rats. This is a very different model from the genetically modified mouse model, in which constitutive expression of Wnt16 was elevated in bone over time v. the burst exposure to rhWnt16b achieved by injection [17, 19, 48, 57]. Additionally, Wnt16 increased cortical bone in mice via increased OPG, primarily affecting early-stage osteoblast lineage cells in the periosteum and not more mature osteocytes, as well as by downregulating osteoclasts [30, 58]. In our study, we tested the ability of rhWnt16b to overcome impaired bone quality and not promotion of bone healing. Three-point bending was used to assess bone quality, which may have led to fractures away from the injection site, resulting in the capture of the whole bone response compared to the localized area of injection [3]. However, when comparing botox injection to other models of limb disuse and cortical thinning such as casting, hindlimb suspension, and nerve ligation, botox injection has shown be the most adaptable method to paralyze the hindlimb with lower incidences of complications such as cast removal by the animal, fluid shift during suspension, or serious adverse events following nerve ligation [18, 22, 34].

Assessment of osseointegration, which involves osteogenesis similar to bone formation during fracture healing, also failed to show an effect of rhWnt16b as a therapeutic agent. Ti implants were placed unicortically so that both cortical bone and trabecular bone could be monitored. Treatment with rhWnt16b increased cortical BIC in botox-treated rats. This suggests Wnt16 regulates cellular responses to maintain a specific threshold of cortical thickness after more severe trauma such as implant placement and subsequent healing. Although rhWnt16b increased bone marrow-to-implant contact in normal rats, it did not affect this parameter in the botox-treated femurs, consistent with the observations that Wnt16 was shown to have limited effects on trabecular bone developmentally [25, 30, 48]. Alveolar bone and femoral bone are different [64]; however, the effect of Wnt16 on cortical bone can be important in clinical dentistry by enhancing BIC in the cortical bone both during implant insertion and, later, during function of the implant in alveolar bone.

Mechanical torsion testing of the osseointegrated implants showed that the increased BIC was not able to rescue the effect of botox on impaired osseointegration, which is consistent with the in vitro analysis showing lower production of osteogenic factors. Collectively, these findings suggest that the mineralized tissue adjacent to the implant was not fully mature and load sustaining.

Clinically, implants with biomimetic implant surfaces may have the potential to temporally regulate and promote the production of both proliferative and differentiation factors to promote cortical bone formation, implant stability, and osseointegration. Factors such as Wnt16 likely contribute by regulating the balance between generating a proliferative pool of pre-osteoblasts and their osteoblastic differentiation due to factors such as sema3a, BMP2, and BMP4. The result is to increase net bone formation.

## Conclusions

Osseointegration is a complex biological cascade that regulates both trabecular and cortical bone regeneration during implant placement. Implants possessing complex multiscale surface topographies augment this regenerative process through the regulation of bone MSCs and later osteoblasts that are in contact with the implant surface. These cells undergo a differentiation process mediated by microroughness and surface wettability and their effects on adsorbed surface proteins. Wnt16 production was shown to be regulated by surface roughness and wettability and acts in accordance with previous literature, increasing OPG production to maintain or increase cortical bone thickness. In this study, we show that rhWnt16b treatment increases total DNA content and proliferative capacity of bone MSCs while reducing differentiation and the production of local factors necessary for mature osteogenesis. This effect is likely independent of other osteogenic pathways like Wnt11-Wnt5a or sema3a signaling. Wnt16 injections did not alter whole bone morphology significantly but were effective at increasing cortical BIC during impaired osseointegration using an established model of impaired osseointegration. However, the mechanical quality of the increased bone was not sufficient to rescue the deleterious effects of the inactivity and nerve signaling loss. Clinically, these results are important to understand the interaction of the cortical and trabecular (cancellous) bone compartments during implant integration and strategies to temporally regulate Wnt16 after implant placement could increase the net pool of progenitor cells able to differentiate into bone-forming osteoblasts leading to improved osseointegration, especially in patients with decreased implant-stabilizing bone volume such as in aging-associated osteoporosis.

Recent work in several fields of science has identified a bias in citation practices such that papers from women and other minority scholars are undercited relative to the number of papers in the field. We recognize this bias and have worked diligently to ensure that we are referencing appropriate papers with fair gender and racial author inclusion.

### Supplementary Information

Below is the link to the electronic supplementary material.

Supplemental Figure 1 Schematic presents the timeline of the animal studies (TIF 130 KB)

Supplemental Figure 2 Treatment of bone marrow stromal cells with Wnt16 to determine the effective dose based on OPG production. (A) Total DNA content was determined in the cell layer lysate after 7 days of culture with either 100 ng/mL or 1 μg/mL of rhWnt16b treated on day 7 or continuously. (B) Osteoprotegerin production was quantified in the conditioned media after 24 h on day 7. Letters on each bar indicate significant differences among groups. Groups that do not share the same letters are significant at p < 0.05; n = 6 per culture/variable (TIF 151 KB)
