# Supplementary figures and images for: Wnt16 Increases Bone-to-Implant Contact in an Osteopenic Rat Model by Increasing Proliferation and Regulating the Differentiation of Bone Marrow Stromal Cells
Source: Ann Biomed Eng. 2024 Mar 22;52(6):1744–62. doi: 10.1007/s10439-024-03488-y (PMC11082046; doi:10.1007/s10439-024-03488-y)

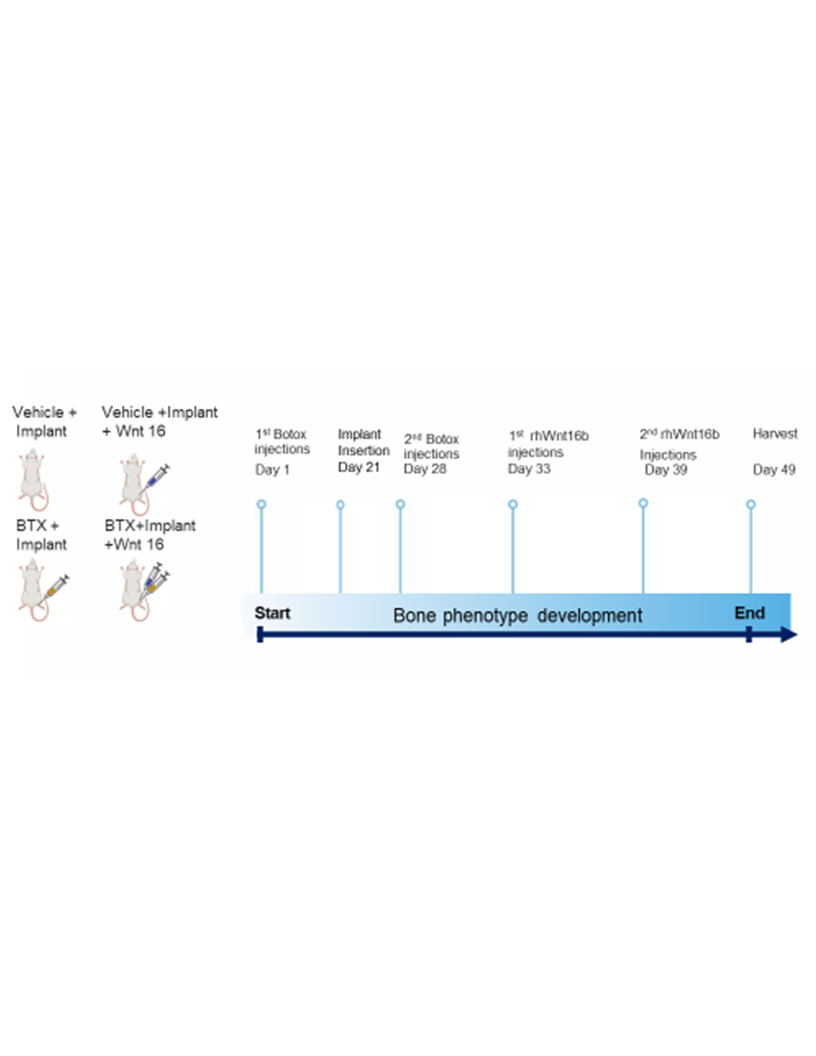

Supplement: Supplementary file 1 — Supplemental Figure 1 Schematic presents the timeline of the animal studies (TIF 130 KB) [file 10439_2024_3488_MOESM1_ESM.tif]

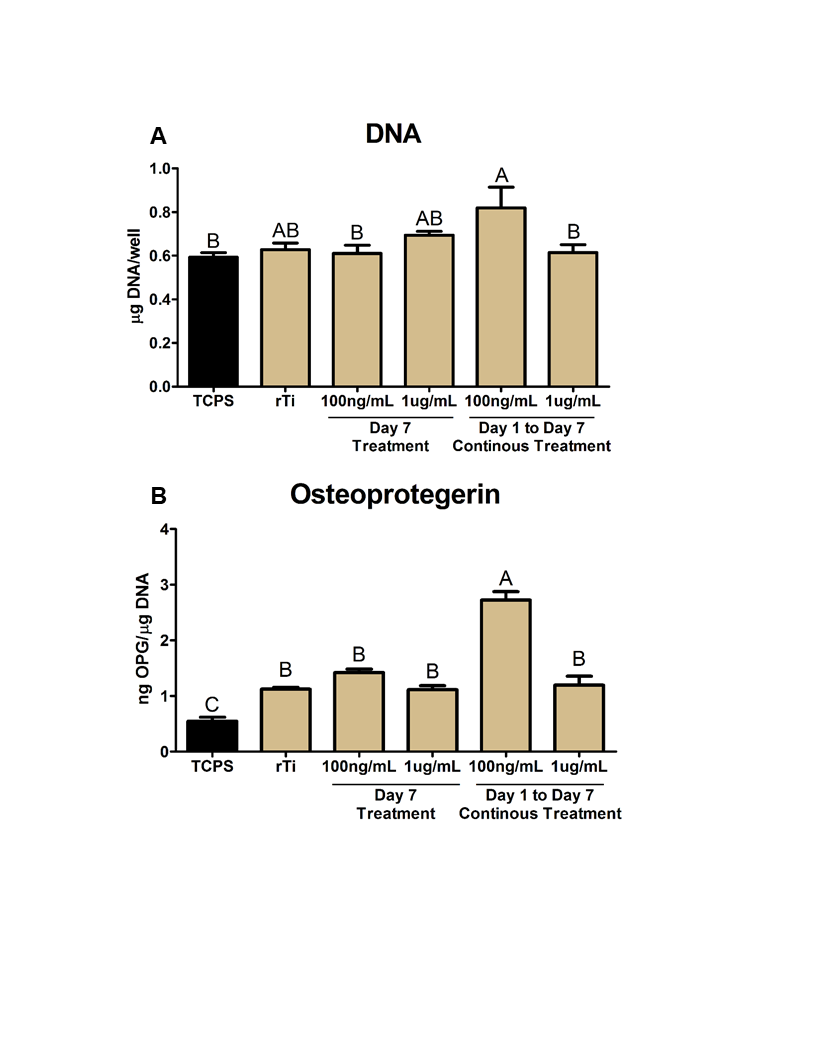

Supplement: Supplementary file 2 — Supplemental Figure 2 Treatment of bone marrow stromal cells with Wnt16 to determine the effective dose based on OPG production. (A) Total DNA content was determined in the cell layer lysate after 7 days of culture with either 100 ng/mL or 1 μg/mL of rhWnt16b treated on day 7 or continuously. (B) Osteoprotegerin production was quantified in the conditioned media after 24 h on day 7. Letters on each bar indicate significant differences among groups. Groups that do not share the same letters are significant at p < 0.05; n = 6 per culture/variable (TIF 151 KB) [file 10439_2024_3488_MOESM2_ESM.tif]
